# From In Vitro Antimicrobial Activity to Food Applications: Limitations of Essential Oils in Real Food Systems

**DOI:** 10.3390/foods15132314

**Published:** 2026-06-29

**Authors:** Ralitsa Kyuchukova

**Affiliations:** Faculty of Veterinary Medicine, Trakia University, 6000 Stara Zagora, Bulgaria; ralitsa.kyuchukova@trakia-uni.bg; Tel.: +359-42699695

**Keywords:** essential oils, antimicrobial activity, food preservation, food matrix, antimicrobial efficacy, biofilms, physicochemical stability, encapsulation, active packaging

## Abstract

Essential oils have attracted considerable attention as natural antimicrobial agents for food preservation due to their broad-spectrum activity against foodborne microorganisms. Although numerous studies report strong antimicrobial effects under in vitro conditions, their effectiveness in real food systems is often substantially reduced. This review critically examines the discrepancy between in vitro antimicrobial activity and actual performance in food matrices. Particular attention is given to the influence of food matrix interactions, physicochemical instability, volatility, sensory limitations, and microbial adaptation on the efficacy of essential oils. A conceptual framework is presented to systematically summarize the major factors limiting antimicrobial performance in practical food applications. In addition, current strategies aimed at improving applicability, including encapsulation technologies, nanoemulsions, synergistic combinations, and active packaging systems, are discussed. Available evidence indicates that simplified experimental models frequently overestimate the practical efficacy of essential oils. More realistic and system-oriented evaluation approaches are therefore necessary to improve the translation of laboratory findings into food applications. Overall, essential oils remain promising candidates for natural food preservation, although their successful industrial application will depend on overcoming important technological and practical limitations.

## 1. Introduction

Consumer demand for minimally processed foods with fewer synthetic preservatives has substantially increased interest in alternative antimicrobial approaches. In particular, the increasing prevalence of foodborne pathogens and the emergence of antimicrobial resistance have raised significant concerns regarding food safety and public health. As a result, there is a strong interest in identifying natural compounds capable of inhibiting microbial growth while maintaining product quality [1,2].

Among these, essential oils have attracted considerable attention due to their complex chemical composition and broad-spectrum antimicrobial activity. These volatile mixtures, primarily composed of terpenes, phenolics, and other bioactive compounds, have been widely investigated for their effectiveness against bacteria, yeasts, and molds [1,3]. Numerous studies have demonstrated that essential oils can exert strong antimicrobial effects under in vitro conditions, often at relatively low concentrations [2,3].

While laboratory-based assays provide controlled and reproducible conditions for evaluating antimicrobial properties, they do not fully reflect the complexity of real food systems. In food matrices, numerous physicochemical and biological factors can influence the availability and activity of essential oil constituents, often resulting in lower antimicrobial efficacy than that observed under in vitro conditions [4,5].

In real food environments, interactions with lipids, proteins, and carbohydrates may reduce the availability of active compounds, while environmental factors such as pH, temperature, and water activity further modulate their antimicrobial performance. In addition, the high volatility and chemical instability of essential oils can lead to rapid loss of activity during processing and storage. Sensory limitations also represent a critical constraint, as concentrations required for microbial inhibition often exceed acceptable thresholds for consumer acceptability and may cause undesirable alterations in flavor and aroma [6,7,8].

These limitations highlight the importance of evaluating essential oils under realistic food conditions rather than relying exclusively on in vitro assays. Rather than focusing solely on their in vitro efficacy, it is essential to understand the factors that limit their performance in real food systems and to identify strategies that can improve their technological feasibility in food preservation [6,7,8].

The aim of this review is to critically analyze the gap between in vitro antimicrobial activity and real-world performance of essential oils in food systems. Special attention is given to the mechanisms responsible for reduced efficacy in complex food matrices.

Furthermore, this review presents a conceptual framework that summarizes the principal factors limiting the efficacy of essential oils in food systems and discusses current strategies aimed at improving their effectiveness in food preservation.

## 2. In Vitro Antimicrobial Activity: Strengths and Limitations

In vitro assays remain among the most commonly applied approaches for assessing the antimicrobial activity of essential oils and represent a fundamental step in screening their potential as natural preservatives. Commonly employed techniques include disk diffusion assays, broth dilution methods for determining minimum inhibitory concentration (MIC), and minimum bactericidal concentration (MBC) assays. These methods provide controlled experimental conditions and facilitate comparison between different essential oils and target microorganisms [9,10]. Because of their simplicity, affordability, and rapid execution, these assays continue to be widely applied as preliminary screening tools for evaluating the antimicrobial potential of newly investigated essential oils and individual bioactive compounds [11,12].

When tested under controlled laboratory conditions, essential oils frequently demonstrate strong antimicrobial activity against a broad spectrum of microorganisms, including Gram-positive and Gram-negative bacteria, as well as yeasts and molds. The antimicrobial activity of essential oils is mainly attributed to bioactive compounds capable of disrupting cell membranes, interfering with enzyme systems, and altering cellular permeability [6,10]. Several constituents, including thymol, carvacrol, eugenol, cinnamaldehyde, and menthol, are considered among the most active antimicrobial compounds due to their ability to destabilize membranes, promote leakage of intracellular materials, and impair essential metabolic functions in microbial cells [5,6,7]. Some essential oils also interfere with quorum sensing mechanisms and biofilm formation, thereby affecting microbial communication and virulence [8,13,14].

However, several important limitations should be considered when interpreting these findings. One of the most critical issues is that these assays are conducted in homogeneous and nutrient-rich media that do not reflect the structural and compositional complexity of real food systems. In such environments, essential oil components are uniformly dispersed and readily available to interact with microbial cells, which frequently results in overestimation of antimicrobial performance [15,16]. In contrast, real food matrices represent heterogeneous environments in which lipids, proteins, carbohydrates, salts, and water may substantially alter the distribution, solubility, and antimicrobial availability of essential oil compounds [15,17]. As a result, antimicrobial concentrations effective under laboratory conditions often demonstrate substantially lower activity when applied to actual food products.

Methodological factors themselves can also introduce variability and bias. The use of organic solvents or emulsifiers to improve the dispersion of hydrophobic essential oils in aqueous media can influence antimicrobial outcomes. Differences in inoculum size, incubation conditions, and endpoint determination further contribute to inconsistencies between studies, limiting comparability and reproducibility [16,18]. Variability may also arise from differences in essential oil composition associated with plant origin, harvest conditions, extraction methods, and storage stability. Substantial variation in essential oil composition associated with botanical origin and environmental conditions may further complicate reproducibility between studies [1,18].

Another important limitation is the focus on planktonic microbial cells, whereas in real food systems microorganisms often exist in more complex forms, such as biofilms or mixed communities. These structured microbial systems exhibit increased resistance to antimicrobial agents, which is not adequately captured by standard in vitro assays [19]. Microorganisms embedded within biofilms are frequently protected by extracellular polymeric matrices that reduce antimicrobial penetration and contribute to increased tolerance toward environmental stress and antimicrobial compounds [8,14,20]. Interactions within mixed microbial communities may also alter susceptibility patterns and microbial behavior during food storage.

Standard in vitro methods also fail to account for critical factors such as volatility, oxidation, and interactions with external variables, all of which can significantly affect the stability and activity of essential oil components in real conditions. Consequently, concentrations appearing highly effective in vitro are often insufficient to achieve comparable antimicrobial effects in food systems [15,18,21]. Conditions encountered during food processing and storage, including temperature fluctuations, oxygen exposure, light, and variations in water activity, can accelerate degradation or evaporation of volatile compounds and progressively reduce antimicrobial performance over time [18,21,22,23].

Taken together, these limitations indicate that standard in vitro assays provide only partial insight into the antimicrobial behavior of essential oils in real food systems. Although these methods remain valuable for preliminary screening and mechanistic evaluation, they cannot fully reproduce the physicochemical and microbiological complexity encountered during food processing and storage. Increasing attention is therefore being directed toward more representative experimental models aimed at reproducing the complexity of real food environments more effectively and improving estimation of antimicrobial performance under industrial conditions [15,17,24].

## 3. The In Vitro–Food System Gap: A Conceptual Framework

The discrepancy between antimicrobial activity observed in vitro and the actual performance of essential oils in food systems represents a critical challenge in their practical application. While numerous studies report strong inhibitory effects under laboratory conditions, these results are often not reproducible in real food environments. This discrepancy emphasizes the need for a more integrative approach to understanding the factors that limit the efficacy of essential oils in complex matrices [15,18]. Recent studies increasingly suggest that antimicrobial behavior in food systems reflects the combined influence of physicochemical, microbiological, and environmental interactions rather than only the intrinsic activity of essential oils [3,4,25].

To facilitate discussion of these discrepancies, a conceptual framework was developed based on factors repeatedly identified in the literature as limiting essential oil efficacy in food systems. A conceptual framework based on five major barriers affecting essential oil efficacy in food systems is presented: (i) partitioning effects, (ii) binding interactions, (iii) physicochemical instability, (iv) sensory constraints, and (v) microbial adaptation. The framework links laboratory observations with technological and biological limitations encountered in practical food preservation systems.

Partitioning effects arise from the hydrophobic nature of essential oil components, which preferentially distribute into lipid phases within food matrices. This reduces their concentration in the aqueous phase, where most microorganisms are located, thereby reducing their effective antimicrobial concentration around microbial cells. This effect is particularly pronounced in high-fat foods, where the efficacy of essential oils is often significantly diminished [15]. Studies investigating meat, dairy, and emulsion-based food systems have demonstrated that lipid-rich environments may substantially reduce the antimicrobial activity of essential oil compounds by decreasing their accessibility to microbial targets [17,26,27]. The extent of partitioning also varies according to the chemical composition of individual essential oils and the physicochemical characteristics of the food matrix itself.

Binding interactions further reduce antimicrobial activity through the association of essential oil compounds with food macromolecules, such as proteins and carbohydrates. These interactions may immobilize active compounds, reducing their bioavailability and limiting effective contact with microbial cells [15,24]. Protein-rich systems are particularly important in this regard, since hydrophobic interactions and non-covalent binding mechanisms may significantly limit the diffusion of antimicrobial constituents within the food environment [15,17]. Comparable effects have been reported in polysaccharide-rich matrices, where viscosity and structural complexity may further restrict compound mobility and microbial exposure.

Another important factor affecting essential oil efficacy is physicochemical instability. Essential oils are highly volatile and susceptible to degradation through oxidation, light exposure, and temperature fluctuations. These processes can lead to the loss or transformation of active constituents, resulting in reduced antimicrobial potency during food processing and storage [18,21]. Thermal treatments, oxygen exposure, and prolonged storage may alter the chemical composition of essential oils through oxidation or evaporation of volatile compounds, thereby reducing both antimicrobial effectiveness and oxidative stability [22,23]. Instability-related changes can also differ considerably among essential oils depending on their dominant constituents and storage conditions.

Sensory properties also limit the practical application of essential oils in foods. While higher concentrations may be required to achieve antimicrobial effects comparable to those observed in vitro, such levels are often unacceptable due to strong flavors and aromas that negatively impact consumer acceptance [16,28]. This issue is particularly relevant in foods with mild sensory characteristics, including dairy products, beverages, seafood, and ready-to-eat products, where even relatively low concentrations of essential oils may substantially modify taste and aroma characteristics [25,26,29]. As a consequence, sensory acceptability often becomes one of the major limitations for the industrial application of essential oils despite promising antimicrobial efficacy under laboratory conditions.

In addition, microbial adaptation may further reduce the effectiveness of essential oils in real food environments. In food environments, microorganisms may form biofilms, exhibit stress responses, or exist within mixed microbial communities, all of which contribute to increased resistance compared to planktonic cells typically used in in vitro assays [19]. Biofilm-associated microorganisms are often protected by extracellular polymeric matrices that reduce antimicrobial penetration and facilitate increased tolerance to environmental stress [8,14,20]. Exposure to sublethal concentrations of essential oils can additionally trigger adaptive stress responses, including changes in membrane composition, activation of efflux systems, and modulation of metabolic activity, which can collectively reduce antimicrobial susceptibility [8,13,14].

These barriers rarely act independently within complex food systems. Partitioning into lipid phases may occur simultaneously with protein binding, while environmental conditions influence both chemical stability and microbial susceptibility. As a result, antimicrobial performance is determined by multiple interacting factors rather than by a single dominant limitation [15,18]. Such interactions may also explain the substantial variability observed between studies using different food matrices, microbial targets, and storage conditions.

**Figure 1:**
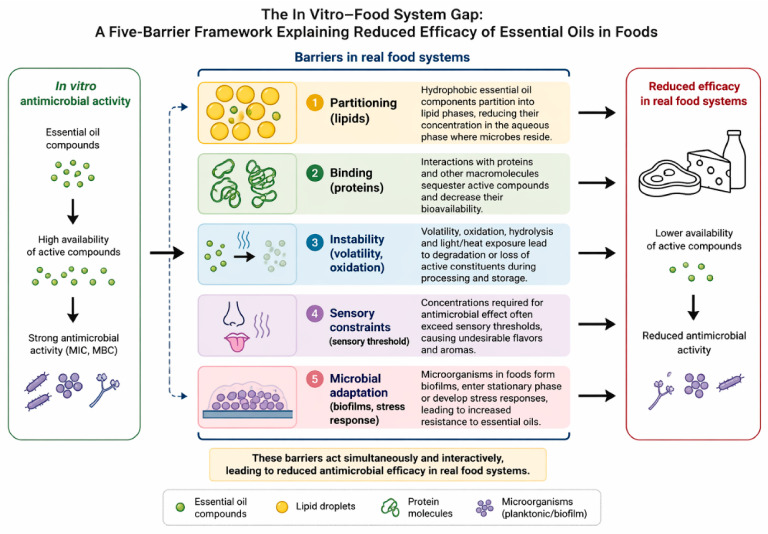
Conceptual framework illustrating the gap between in vitro antimicrobial activity and efficacy in real food systems, based on literature findings regarding food matrix interactions, physicochemical instability, sensory limitations, and microbial adaptation [15,18,19,28].

Figure 1 summarizes the principal barriers responsible for the reduced antimicrobial efficacy of essential oils in food systems and shows how these factors interact within complex food matrices. The figure illustrates why antimicrobial activity observed under in vitro conditions cannot always be directly translated into effective performance in real food systems.

The proposed framework offers a practical approach for interpreting discrepancies between laboratory findings and antimicrobial performance in real food systems. Identification of the principal barriers affecting efficacy may facilitate development of more effective preservation strategies and experimentally relevant food models for future studies.

The major barriers affecting the antimicrobial efficacy of essential oils in food systems and their associated mechanisms are summarized in Table 1.

## 4. Factors Limiting Efficacy in Real Food Systems

### 4.1. Food Matrix Effects

The composition and structural complexity of food matrices are among the major factors influencing the antimicrobial efficacy of essential oils. Unlike simplified laboratory media, food systems contain heterogeneous mixtures of lipids, proteins, carbohydrates, and water, all of which may interact with essential oil components and modify their activity [15,18]. Therefore, antimicrobial efficacy depends not only on the chemical composition of essential oils but also on the physicochemical properties of the food matrix in which they are incorporated. As a result, antimicrobial activity observed under laboratory conditions is often not reproduced to the same extent in real food systems [3,15,17].

Food matrix components may substantially reduce the availability of antimicrobial compounds through mechanisms such as lipid partitioning and interactions with proteins and polysaccharides. These interactions may limit compound mobility and reduce effective contact with microbial cells, particularly in high-fat and protein-rich foods [15,17,24,27]. In addition, the physical structure of food matrices may hinder the uniform distribution of essential oils, leading to localized variations in antimicrobial activity.

Water activity and pH may additionally influence the interaction between essential oil compounds and food components. Variations in these parameters can alter compound solubility, microbial susceptibility, and the physicochemical stability of essential oil constituents, thereby contributing to variability in antimicrobial outcomes among different food products [15,18]. Furthermore, the structural heterogeneity of real foods can create localized microenvironments in which microorganisms experience different levels of exposure to antimicrobial compounds, making microbial inhibition less predictable than under homogeneous laboratory conditions.

Taken together, these interactions demonstrate that the food matrix strongly influences the distribution, availability, and antimicrobial effectiveness of essential oil compounds in real food systems. These observations also highlight the limitations of relying exclusively on simplified in vitro assays that do not adequately reproduce the physicochemical and structural complexity of foods. Improved understanding of matrix-related interactions is important for developing more effective and technologically feasible preservation strategies [15,17,24,30].

Variability in essential oil composition represents an additional factor influencing antimicrobial performance. The relative concentration of active compounds may differ substantially depending on plant species, geographical origin, climatic conditions, harvest season, extraction method, and storage conditions [1,4,18]. Consequently, essential oils derived from the same botanical source may exhibit considerable differences in antimicrobial activity between studies. Such variability complicates standardization and makes direct comparison between studies more difficult, creating additional challenges for industrial implementation of essential oil-based preservation systems [11,18].

### 4.2. Physicochemical Instability

The physicochemical characteristics of essential oils, particularly their volatility and susceptibility to degradation, represent important limitations for their practical application in food systems. Because essential oils contain highly volatile compounds, significant losses may occur during food processing, storage, and handling, resulting in reduced antimicrobial activity [18,21]. This issue becomes particularly relevant during thermal processing operations, including pasteurization, drying, cooking, and extrusion, where elevated temperatures may accelerate evaporation of volatile constituents and reduce the concentration of biologically active compounds remaining in the final product [22]. In addition, prolonged storage and repeated exposure to air may further intensify the loss of volatile fractions, thereby reducing the long-term antimicrobial effectiveness of essential oils in food systems.

In addition to volatility, essential oil components are susceptible to oxidative and photochemical degradation. Exposure to oxygen, light, and elevated temperatures may alter their chemical composition and reduce antimicrobial activity during processing and storage [16,21,31]. Such changes may also affect aroma profile, color stability, and overall product quality [18,29]. The extent of degradation depends largely on the chemical composition of individual essential oils, as some constituents exhibit greater stability than others under comparable environmental conditions. For example, phenolic compounds such as thymol and carvacrol are generally more stable than highly volatile monoterpenes [5,6].

Environmental conditions such as pH and water activity may further influence the stability and antimicrobial performance of essential oil constituents. Because these parameters vary considerably among food systems, they can affect compound solubility, microbial susceptibility, and interactions with food components [15,18]. Consequently, antimicrobial efficacy should always be evaluated within the physicochemical context of the specific food matrix under investigation.

Packaging conditions and storage atmosphere also strongly influence essential oil stability. Exposure to oxygen-rich environments may accelerate oxidative degradation, while inappropriate packaging materials may permit migration or evaporation of volatile compounds during storage [32,33,34]. Recent studies have therefore increasingly explored encapsulation technologies, nanoemulsion systems, and active packaging approaches aimed at improving the physicochemical stability and controlled release of essential oil constituents in food systems [22,35,36,37,38].

Volatility, oxidative degradation, environmental conditions, and storage-related factors can substantially influence the antimicrobial stability of essential oils in food systems. As a result, antimicrobial performance under real processing and storage conditions is often lower than that observed in simplified in vitro environments. Improving physicochemical stability therefore remains an important prerequisite for the broader application of essential oils in food preservation.

### 4.3. Sensory Constraints

One of the major limitations associated with the use of essential oils in foods is their intense aroma and flavor. Although antimicrobial activity may require relatively high concentrations, such levels are frequently incompatible with consumer preferences because of undesirable sensory alterations [16,28]. This remains an important limitation for industrial application, since antimicrobial effectiveness alone is insufficient if product acceptability is negatively affected [25,29,39].

Essential oils possess distinctive sensory characteristics that may substantially affect the taste, odor, and overall acceptability of food products. This issue is particularly relevant in foods with mild or delicate flavor profiles, including dairy products, beverages, seafood, bakery products, and minimally processed ready-to-eat foods, where even low concentrations may become perceptible [18,26,29,40]. In contrast, strongly flavored products may better tolerate essential oil incorporation, although the extent of tolerance depends on both the food formulation and the specific essential oil used.

As a result, antimicrobial efficacy and sensory acceptability must be carefully balanced. Concentrations that effectively inhibit microbial growth may exceed acceptable sensory thresholds, thereby limiting practical application in food systems [28,40]. Consumer acceptance may further vary according to cultural preferences, geographic regions, and product categories [25,41]. Therefore, sensory evaluation should be considered an integral component of developing essential oil-based preservation strategies rather than a secondary consideration.

Several technological approaches have been investigated to reduce sensory limitations while preserving antimicrobial activity. These include encapsulation technologies, nanoemulsion systems, active packaging applications, and synergistic combinations with other antimicrobial agents that allow lower concentrations of essential oils to be used [22,35,36,37,38]. Controlled-release systems may reduce the immediate sensory impact of essential oils while maintaining antimicrobial activity during storage. In addition, selecting essential oils with milder sensory profiles or adapting formulations to specific food products may further improve consumer acceptability [25,29].

Overall, sensory characteristics remain one of the principal factors limiting the practical application of essential oils in foods. Successful industrial implementation requires balancing antimicrobial efficacy with sensory quality, technological feasibility, and consumer acceptance within the target food system.

### 4.4. Microbial Behavior (Biofilms, Stress Response)

Microbial behavior in food systems differs substantially from that observed under simplified in vitro conditions, which can reduce microbial susceptibility to essential oils. In real food environments, microorganisms frequently exist in structured communities such as biofilms that provide increased protection against antimicrobial compounds. Biofilm formation is particularly important in food processing environments, where microorganisms can colonize food-contact surfaces, processing equipment, and storage areas, allowing them to persist despite antimicrobial treatments [8,14,19]. As a result, microbial populations present in real food systems often exhibit greater tolerance than planktonic cultures typically used in laboratory assays.

Biofilms are characterized by the production of extracellular polymeric substances that form a protective matrix around microbial cells. This matrix limits the penetration of antimicrobial compounds and contributes to increased microbial tolerance [8,14,19,20]. In addition, microorganisms within biofilms often exhibit altered metabolic activity and physiological heterogeneity, which may further reduce susceptibility to antimicrobial stress compared with planktonic cells.

Exposure to sublethal concentrations of essential oils may also activate stress responses that improve microbial survival. These responses can include changes in membrane composition, activation of efflux pumps, production of protective enzymes, and modulation of metabolic activity [8,13,14,20]. Consequently, microorganisms exposed to prolonged sublethal stress may become less susceptible to antimicrobial treatments.

Mixed microbial populations present in food systems can further influence antimicrobial outcomes because interactions between microbial species may increase overall resilience. Such interactions may affect nutrient availability, quorum sensing activity, extracellular matrix production, and collective stress responses, all of which can modify susceptibility to antimicrobial compounds [8,13]. Consequently, microbial behavior in complex food environments may differ substantially from that observed in single-species laboratory cultures [8,19].

Another important aspect involves differences in susceptibility among microbial groups. Gram-negative bacteria often exhibit greater tolerance toward essential oils because of the structural complexity of their outer membrane, whereas Gram-positive bacteria may be more susceptible to membrane-disrupting compounds [6,10]. However, susceptibility patterns can vary considerably depending on the chemical composition of the essential oil, microbial species, environmental conditions, and the presence of protective food matrix components.

The persistence of biofilms and stress-adapted microbial populations remains a significant food safety concern because these systems may contribute not only to reduced antimicrobial efficacy but also to long-term contamination during food processing and storage. For this reason, recent studies have increasingly explored combined preservation strategies, biofilm-targeted approaches, and controlled-release antimicrobial systems designed to improve essential oil performance under realistic food-processing conditions [14,35,36,42].

The practical consequences of the major barriers affecting essential oil efficacy in food systems are summarized in Table 2.

### 4.5. Comparative Analysis of Essential Oil Performance in Different Food Systems

The antimicrobial activity of essential oils observed under in vitro conditions is frequently lower when these compounds are applied in real foods. Although many studies have reported low MIC and MBC values against foodborne microorganisms, the same essential oils often show reduced effectiveness after incorporation into food products [19,43]. This discrepancy between laboratory findings and food applications remains one of the main obstacles to the wider use of essential oils as natural preservatives.

The extent of this reduction varies among food categories. Meat products generally represent one of the most challenging matrices because antimicrobial efficacy is often diminished after incorporation into the food system. Similar limitations have been reported in dairy products, while in fish and seafood products antimicrobial performance may be further influenced by storage conditions and product-specific sensory requirements [43,44,45,46].

Published studies consistently demonstrate that EO concentrations capable of inhibiting microbial growth under laboratory conditions do not necessarily produce the same effect in foods. Consequently, higher concentrations are often required to achieve measurable microbial reductions. However, increasing EO concentration may negatively affect sensory quality and consumer acceptance [43,46].

The studies presented in Table 3, Table 4 and Table 5 compare EO performance in meat, dairy, and fish products and illustrate how food characteristics influence antimicrobial efficacy. Together, these studies demonstrate that successful application of essential oils depends not only on their intrinsic antimicrobial activity but also on the specific conditions of the food system in which they are used.

#### 4.5.1. Essential Oils in Meat and Meat Products

Among the food categories investigated to date, meat and meat products are frequently reported as particularly challenging matrices for the practical application of essential oils. Although strong antimicrobial activity is often observed under laboratory conditions, substantially lower efficacy is commonly reported after incorporation into meat products [19,47,49]. This discrepancy highlights the difficulty of translating promising in vitro findings into practical food preservation applications.

The studies presented in Table 3 illustrate this challenge. Essential oils exhibiting low MIC values against pathogens such as Listeria monocytogenes and Yersinia enterocolitica often achieved only limited microbial reductions when applied to minced meat systems. In several cases, measurable antimicrobial effects were observed only at concentrations exceeding those predicted from laboratory assays, while some treatments failed to produce significant reductions despite promising in vitro activity [19,47,49].

Overall, available evidence indicates that antimicrobial performance in meat products is strongly influenced by the characteristics of the food system itself. Consequently, results obtained under laboratory conditions may not accurately predict efficacy in meat matrices, emphasizing the importance of evaluating essential oils directly in food applications when assessing their preservation potential [48,49].

#### 4.5.2. Essential Oils in Dairy Products

Dairy products represent a particularly challenging environment for the application of essential oils as antimicrobial agents. Although many essential oils demonstrate strong antimicrobial activity under laboratory conditions, their effectiveness is often reduced when applied to milk, cheese, yogurt, and other dairy products [43,44,50,52,53]. As in other complex food systems, antimicrobial performance in dairy products is influenced by multiple matrix-related factors that can limit the activity of essential oil constituents.

The influence of the dairy matrix is particularly evident in cheese and fermented dairy products. In these systems, antimicrobial efficacy is often lower than expected from laboratory studies, and the extent of microbial inhibition may vary considerably among products [43,44,50]. Additional factors such as product composition, starter cultures, storage conditions, and microbial interactions can further influence antimicrobial performance.

A recent study by Kayiran et al. demonstrated that *Thymbra spicata* essential oil produced inhibition zones exceeding 30 mm against several bacterial species under laboratory conditions, whereas nanoemulsion formulations containing the same EO failed to exhibit measurable antibacterial activity despite contributing to delayed spoilage during cheese storage. These findings illustrate that technological modifications designed to improve stability may simultaneously alter the immediate antimicrobial performance observed in vitro [50].

Similarly, Doukaki et al. reported only moderate antimicrobial activity when 0.5% oregano essential oil was incorporated into sodium alginate edible films and applied to Feta cheese. No significant reduction was observed against *E. coli* O157, whereas only partial inhibition of Listeria monocytogenes was achieved. Nevertheless, the treatment extended product shelf life under refrigerated storage conditions. These findings demonstrate that highly active essential oils may perform less effectively in dairy products than would be predicted from laboratory assays [43].

In contrast, Liu et al. reported that oregano essential oil effectively delayed microbial growth in pasteurized milk, with the highest tested concentration (2.0 mg/L) maintaining total microbial counts below the regulatory limit throughout eight days of refrigerated storage [44]. This observation suggests that the effectiveness of essential oils may vary considerably among dairy products depending on product characteristics and storage conditions.

Another important consideration is sensory acceptability. Because many dairy products possess relatively mild flavor profiles, even low concentrations of essential oils may noticeably alter aroma and taste, thereby limiting the concentrations that can be applied in practice [43,50].

The studies summarized in Table 4 illustrate the variability of essential oil performance across different dairy products. Collectively, these findings demonstrate that successful application of essential oils in dairy systems depends not only on their antimicrobial activity but also on product characteristics, processing conditions, and consumer acceptability.

#### 4.5.3. Essential Oils in Fish and Seafood Products

Fish and seafood products are highly susceptible to microbial spoilage and oxidative deterioration, making them attractive targets for the application of natural preservatives such as essential oils. Numerous studies have reported antimicrobial and antioxidant effects of oregano, thyme, rosemary, clove, and cinnamon essential oils in fish products; however, their effectiveness under real storage conditions is often lower than that observed in laboratory assays [45,46,54,55,56].

Fish products present unique preservation challenges because microbial spoilage and lipid oxidation occur simultaneously during storage. Consequently, essential oils used in these products are often expected to provide both antimicrobial and antioxidant protection. Several studies have shown that EO application can delay microbial growth, reduce lipid oxidation, and extend product shelf life, although the magnitude of these effects varies considerably depending on the fish species, storage conditions, and delivery system employed [45,56,57].

To improve practical performance, recent research has increasingly focused on edible coatings, active packaging materials, and nanoemulsion systems designed to enhance the stability and controlled release of essential oil constituents [45,56,57].

Van Haute et al. reported that immersion of salmon and scampi in marinades containing oregano, thyme, or cinnamon essential oils reduced the growth of spoilage microorganisms and extended microbial shelf life. However, the concentrations required to achieve measurable antimicrobial effects also influenced sensory properties, highlighting an important limitation associated with EO application in fish and seafood products [46].

Recent research has further demonstrated the potential of oregano essential oil incorporated into edible coatings for fish preservation. Rosati et al. developed an alginate-based emulsion containing 1% oregano essential oil and applied it to frozen–thawed ready-to-cook hake filets. The coating effectively suppressed the growth of spoilage microbiota, reduced lipid oxidation, and improved product quality during refrigerated storage [45].

Similar findings were reported by Wang et al., who incorporated wampee seed essential oil into a chitosan-based edible film combined with cold plasma treatment and modified atmosphere packaging for refrigerated storage of golden pompano filets. The combined preservation strategy significantly reduced bacterial growth, delayed lipid oxidation, and improved quality retention throughout storage, demonstrating the potential of multi-hurdle approaches for enhancing EO performance in fish products [58].

In a recent review, Pierozan et al. [59] emphasized that successful application of essential oils in fish and seafood products depends not only on antimicrobial activity but also on maintaining product quality and sensory acceptability during storage and processing.

The studies summarized in Table 5 provide representative examples of EO applications in fish and seafood products and illustrate both the potential and the limitations of translating promising in vitro antimicrobial activity into effective food preservation strategies.

**Table 5:** Representative examples of essential oil applications in fish and seafood products and their impact on microbial stability and shelf-life extension.

EO/Active Compound	Delivery System	Food Matrix	Main Findings in Food System	Main Limitation	Ref.
Oregano, thyme, cinnamon EOs	Marinade immersion	Salmon and scampi	Spoilage microbiota ↓; shelf life extended during refrigerated storage	Sensory constraints at effective doses	[46]
Carvacrol + EGCG	Fish gelatin edible coating	Sea bass filets (*Lateolabrax japonicus*)	*P. aeruginosa*, H_2_S-producing bacteria, yeasts and molds ↓; TVB-N and TBA ↓; shelf life improved	Multi-component coating required	[56]
WSEO	Chitosan film + cold plasma + MAP	Golden pompano filets (*Trachinotus blochii*)	Microbial growth ↓; TVB-N and lipid oxidation ↓; quality retention improved	Multi-hurdle system; EO contribution difficult to isolate	[58]
Oregano EO	Alginate emulsion coating	Frozen–thawed hake filets	*Pseudomonas* spp. suppressed; mesophilic counts ↓; lipid oxidation ↓; sensory quality improved	Sensory impact requires further evaluation	[45]
Thymol	Alginate coating with NE and NLC	White shrimp (*Litopenaeus vannamei*)	Microbial growth, TVB-N, TBA and pH ↓; shelf life extended from 4 to 10 d	Nanoencapsulation required	[57]

**Abbreviations:** EO, essential oil; EGCG, epigallocatechin gallate; WSEO, wampee seed essential oil; MAP, modified atmosphere packaging; TVB-N, total volatile basic nitrogen; TBA, thiobarbituric acid index; NE, nanoemulsion; NLC, nanostructured lipid carriers; ↓, reduction.

## 5. Bridging the Gap: Strategies for Real Applications

### 5.1. Encapsulation and Nanoemulsions

Among the strategies currently investigated to overcome the limitations associated with essential oils in food systems, encapsulation and nanoemulsion technologies have attracted considerable interest. These approaches are designed to improve the stability, dispersion, and practical performance of essential oil components in complex food matrices [22,23,37,38]. By protecting bioactive compounds from environmental degradation and facilitating their incorporation into food systems, such technologies may help overcome several limitations associated with the direct use of essential oils.

Encapsulation techniques, including microencapsulation and nanoencapsulation, incorporate essential oils into protective carrier systems that reduce volatility and improve stability during processing and storage. Nanoemulsions, in turn, enhance the dispersion of hydrophobic compounds in aqueous environments and promote more uniform distribution throughout the food matrix [16,26,35,36]. Depending on the carrier materials used, these systems may influence release kinetics, stability, and antimicrobial performance [22,37,38].

Several studies have demonstrated the potential of these technologies in food applications. Kayiran et al. [50] evaluated nanoemulsions containing *Thymbra spicata* essential oil in cheese and observed improved physicochemical stability compared with the non-encapsulated oil. However, despite the improved stability, the nanoemulsion did not produce measurable inhibition zones against the tested microorganisms. Nevertheless, spoilage development was delayed during storage, suggesting that enhanced stability alone may not always result in stronger antimicrobial activity.

Dastaran et al. [57] investigated a thymol nanoemulsion for shrimp preservation during refrigerated storage. The treatment reduced microbial growth, delayed lipid oxidation, and improved quality parameters compared with untreated samples. Similarly, Rosati et al. [45] incorporated oregano essential oil into an alginate coating applied to frozen–thawed hake filets. The coated samples showed lower spoilage microbiota counts, reduced lipid oxidation, and improved sensory quality during storage. Kurek et al. [60] also highlighted the value of edible coating systems as carriers of antimicrobial and antioxidant compounds in fish preservation.

An important advantage of encapsulation-based systems is their ability to provide controlled release of active compounds during storage. This may prolong antimicrobial activity and reduce the need for high initial concentrations of essential oils, which is particularly relevant in food systems sensitive to sensory changes [22,36,37]. These findings suggest that encapsulation and nanoemulsion systems may help overcome some of the practical limitations that currently restrict the use of essential oils in food preservation.

Despite these advantages, several technological and industrial limitations remain. Production costs, scalability, long-term stability, compatibility with food matrices, and regulatory considerations may complicate large-scale implementation in commercial food production [35,36,38,41]. In addition, some encapsulation materials and nanostructured systems may require further toxicological evaluation before widespread industrial adoption. Further studies are therefore needed to assess their economic feasibility and practical applicability under commercial production conditions.

### 5.2. Combination Strategies (EO + Antibiotics/Preservatives)

Another effective strategy involves the use of essential oils in combination with other antimicrobial agents, including organic acids, bacteriocins, or conventional preservatives. These combinations may produce synergistic effects against microorganisms, allowing lower concentrations of essential oils to be used while maintaining or enhancing antimicrobial efficacy [16,61,62]. Interest in combination strategies has increased because they may improve microbial control and help address some of the practical limitations associated with the use of essential oils in foods [42,62].

Several mechanisms may contribute to synergistic interactions, including increased membrane permeability, inhibition of resistance mechanisms, disruption of metabolic pathways, and enhanced penetration of antimicrobial compounds into microbial cells [6,8,62]. Some studies have also suggested that combined treatments may interfere with quorum sensing systems and reduce biofilm-associated tolerance, thereby increasing microbial susceptibility to antimicrobial stress [8,13,14,42].

Synergistic preservation approaches may involve combinations of essential oils with organic acids, bacteriocins, mild thermal treatments, modified atmosphere packaging, or conventional preservatives [16,42,62]. However, their effectiveness depends on factors such as food composition, microbial species, storage conditions, and the chemical characteristics of the essential oil used. Consequently, these systems require evaluation under realistic food-processing and storage conditions.

Yu et al. [63] reviewed the combined use of bacteriocins and other antimicrobial compounds and reported that these approaches often provide greater inhibition of foodborne pathogens than individual treatments alone.

Similarly, Bukvicki et al. [64] summarized numerous applications involving essential oils, lactic acid bacteria, and bacteriocins. The authors reported that synergistic interactions frequently allow lower concentrations of antimicrobial agents while maintaining effective microbial control. Enhanced inhibition of *Listeria monocytogenes*, *Salmonella* spp., and other foodborne pathogens was observed when essential oils were combined with bacteriocins such as nisin and enterocins.

Iseppi et al. [65] developed an edible coating containing essential oils and the bacteriocin bacLP17 for the control of *Listeria monocytogenes* in shrimp. A pronounced synergistic effect was observed between the antimicrobial agents. EO–EO combinations reduced the concentrations required for antimicrobial activity by approximately 10–20-fold, whereas EO–bacteriocin combinations reduced the required concentrations by up to 40-fold compared with the individual compounds. The reduced amount of essential oils also helped minimize the intense aroma commonly associated with EO applications.

Wang et al. [58] investigated the combined application of wampee seed essential oil, cold plasma treatment, and modified atmosphere packaging during refrigerated storage of fish filets. Microbial growth and quality deterioration were more effectively controlled in the combined treatment than in samples subjected to the individual preservation methods. These findings further support the use of multi-hurdle preservation systems for improving the practical performance of essential oils in food applications.

Combination strategies may offer several advantages, including improved antimicrobial efficacy, reduced concentrations of individual preservatives, and better compatibility with sensory requirements. At the same time, the complexity of these systems may introduce technological challenges, including possible antagonistic interactions and variability in performance depending on the food matrix and storage conditions.

Overall, the available evidence indicates that combination strategies represent a promising approach for enhancing the effectiveness of essential oils while reducing some of the limitations associated with their direct application in foods [66].

### 5.3. Active Packaging Systems

Active packaging systems incorporating essential oils are increasingly investigated as a strategy for improving antimicrobial performance in food applications. In this approach, essential oils are integrated into packaging materials, enabling gradual release of antimicrobial compounds into the food environment. Compared with direct addition of essential oils to foods, active packaging may provide more controlled antimicrobial activity while limiting interactions between essential oil constituents and food components [21,32,36]. In recent years, these systems have attracted considerable interest because they align with current trends toward sustainable packaging, clean-label products, and reduced use of synthetic preservatives [32,33,41].

Various packaging materials have been investigated for incorporation of essential oils, including biodegradable films, edible coatings, polysaccharide-based systems, protein films, and nanocomposite packaging materials [33,34,36,67,68]. Several studies have shown that incorporation of essential oils into biopolymeric films can enhance antimicrobial and antioxidant properties without substantially affecting the functional characteristics of the packaging material [34,36,67,68,69,70].

The gradual release of essential oil constituents may provide prolonged antimicrobial protection and contribute to shelf-life extension in perishable foods. Applications have been investigated in meat, fish, dairy products, fruits, vegetables, and bakery products, where active packaging systems have demonstrated the ability to reduce microbial growth and delay spoilage during storage [26,34,36]. This approach may be particularly advantageous in meat and seafood products, where direct addition of essential oils can negatively affect sensory quality [26,29].

Doukaki et al. [43] evaluated sodium alginate edible films containing oregano essential oil for preservation of Feta cheese. Although no substantial reduction in *Escherichia coli* O157 was observed, the treatment partially inhibited *Listeria monocytogenes* and extended product shelf life under refrigerated conditions. The results suggest that active packaging systems may provide preservation benefits even when complete microbial inhibition is not achieved.

Rosati et al. [45] incorporated oregano essential oil into an alginate-based coating applied to frozen–thawed hake filets. The coated samples showed lower spoilage microbiota counts, reduced lipid oxidation, and improved sensory quality during refrigerated storage compared with untreated controls. Similarly, Song et al. [56] reported that essential oil-based packaging materials delayed microbial growth and quality deterioration in aquatic food products, while maintaining antimicrobial activity throughout storage.

Overall, available studies indicate that active packaging systems can improve the practical performance of essential oils by enhancing their persistence during storage and reducing some of the limitations associated with direct incorporation into foods.

Despite encouraging results, several challenges continue to restrict broader industrial implementation. These include production cost, scalability, migration control, long-term stability of active compounds, compatibility with packaging technologies, and regulatory approval of packaging materials containing bioactive substances [33,36,41]. In addition, optimization of release kinetics remains essential, since both excessively rapid and insufficient release may compromise antimicrobial efficacy.

Current evidence suggests that active packaging systems represent a promising approach for improving the practical applicability of essential oils in food preservation. However, further research is needed to optimize these technologies and evaluate their performance under commercial production conditions.

### 5.4. Dose Optimization vs. Sensory Acceptability

One of the major challenges associated with the application of essential oils in food systems is balancing antimicrobial efficacy with sensory acceptability. Although in vitro studies often report inhibitory effects at relatively low concentrations, higher doses are frequently required to achieve comparable antimicrobial activity in real food matrices. In many cases, however, these concentrations may negatively affect product aroma, flavor, and overall consumer acceptance [16,18,28,39]. Consequently, identifying effective yet sensorially acceptable concentrations remains a critical aspect of essential oil application in food preservation.

The relationship between antimicrobial efficacy and sensory quality is highly product-specific. Factors such as food composition, storage conditions, target microorganisms, and the chemical profile of the selected essential oil may influence both antimicrobial performance and consumer perception [15,18]. As a result, concentration ranges that are effective in one food product may not be suitable for another.

In many food systems, the concentration required for effective microbial control approaches or exceeds acceptable sensory thresholds. This challenge is particularly evident in highly perishable foods, where strong antimicrobial protection is needed to maintain product safety and shelf life [26,29].

Van Haute et al. [46] reported that marinades containing oregano, thyme, and cinnamon essential oils reduced spoilage microbiota and extended the microbial shelf life of salmon and scampi. However, the concentrations required to achieve these effects also influenced sensory properties, illustrating the practical difficulty of balancing antimicrobial efficacy with consumer acceptance.

Several strategies have been proposed to address this challenge. These include encapsulation technologies, nanoemulsion systems, active packaging applications, and synergistic preservation approaches that reduce the concentration of essential oils required for microbial control [22,35,36,37,38]. Such technologies may improve the practical applicability of essential oils while minimizing undesirable sensory effects.

Another promising approach involves combining essential oils with complementary preservation methods, including bacteriocins, organic acids, mild thermal treatments, or modified atmosphere packaging [42,62]. These combinations may allow lower concentrations of essential oils to be used without compromising antimicrobial performance. Selection of essential oils that are naturally compatible with the sensory profile of specific foods may further improve consumer acceptance.

Dose optimization therefore remains a key factor determining the successful application of essential oils in food preservation. Future research should focus on developing formulation strategies that maintain antimicrobial efficacy while ensuring acceptable sensory quality and technological feasibility.

The main strategies currently used to improve the efficacy and applicability of essential oils in food systems are summarized in Table 6.

## 6. Toward More Realistic Evaluation Models

The limitations of in vitro assays in predicting the antimicrobial efficacy of essential oils in food systems emphasize the need for more realistic and application-oriented evaluation models. Although conventional laboratory methods provide important preliminary information, they often fail to reproduce the complexity of real food environments, which substantially limits their predictive relevance. As a result, increasing attention is being directed toward experimental models that better reflect conditions encountered in real food systems [15,18]. This reflects increasing awareness that antimicrobial efficacy cannot be reliably interpreted using simplified laboratory conditions alone [3,17]. More representative evaluation systems are therefore expected to improve prediction of antimicrobial performance during actual food processing and storage.

One important direction involves the use of model food systems that better reproduce the physicochemical characteristics of real foods. Such systems may include simplified formulations containing relevant components such as lipids, proteins, and carbohydrates, allowing matrix-related effects to be investigated under controlled conditions. Compared with traditional culture media, these models often provide more reliable estimates of antimicrobial performance because they incorporate greater structural and compositional complexity [15,24]. Model food systems also allow investigation of specific interactions between essential oil compounds and individual food components, thereby improving understanding of matrix-related limitations affecting antimicrobial activity [15,17,71]. In addition, these systems make it possible to evaluate the influence of processing variables such as fat content, pH, water activity, and storage conditions under more realistic but still controlled environments.

The inclusion of structured microbial systems is another important aspect that deserves greater attention in antimicrobial testing. Unlike planktonic cells commonly used in in vitro assays, microorganisms in food environments frequently occur in biofilms or mixed-species communities. These microbial systems often exhibit increased resistance and physiological adaptations, both of which may significantly influence antimicrobial outcomes. For this reason, biofilm-based assays and multi-species models represent promising approaches for improving the practical relevance of experimental findings [8,13,19,72]. Such systems more closely reproduce microbial behavior encountered in food processing facilities, storage environments, and food contact surfaces, where biofilm formation frequently contributes to contamination persistence and increased antimicrobial tolerance [8,73]. Interactions between microbial species within mixed communities may additionally alter metabolic activity, stress responses, and susceptibility patterns, making single-species laboratory assays insufficient for predicting antimicrobial performance in real food systems.

Methodological variability between studies also remains a significant challenge. Differences in inoculum size, incubation conditions, and criteria used to determine antimicrobial activity often complicate direct comparison of published results. Greater methodological standardization and clearer reporting practices would likely improve reproducibility and support more reliable interpretation of antimicrobial data [11,12]. Limited interlaboratory reproducibility also represents a major obstacle when comparing antimicrobial efficacy data obtained using different experimental platforms and food systems.

Standardization of essential oil composition and antimicrobial testing protocols remains essential for improving reproducibility and industrial applicability. Variability in chemical profile, extraction procedures, and experimental methodologies may substantially influence reported antimicrobial outcomes and complicate comparison between studies [1,18].

Environmental parameters such as pH, temperature, and water activity should also be integrated more consistently into experimental designs. These factors strongly influence both stability of essential oil compounds and microbial susceptibility, yet they are frequently simplified or insufficiently controlled under laboratory conditions [15,18]. Storage atmosphere, oxygen availability, packaging conditions, and temperature fluctuations during storage may additionally influence antimicrobial performance in real food systems [32,33]. Incorporating these variables into experimental models could improve the predictive value of antimicrobial assessments and better reflect industrial food preservation conditions.

Advanced analytical and modeling approaches may provide additional opportunities for improving antimicrobial evaluation. Techniques such as predictive microbiology, kinetic modeling, and systems-based analysis help describe the dynamic interactions between essential oils, food matrices, and microbial populations. These approaches improve understanding of antimicrobial behavior under realistic conditions and help optimize practical application strategies [36,74]. Mathematical modeling and predictive microbiology can also be used to estimate microbial growth dynamics, antimicrobial diffusion behavior, and shelf-life responses under different storage conditions, allowing more rational design of preservation systems [74]. Beyond conventional predictive models, emerging technologies involving artificial intelligence, machine learning, and data-driven approaches are increasingly being explored for optimization of formulation strategies and prediction of antimicrobial performance in complex food systems [75].

Pilot-scale studies and industrial validation experiments represent another important step toward improving the practical relevance of antimicrobial evaluation. Although laboratory-scale studies provide valuable mechanistic information, industrial food systems involve additional variables related to processing scale, equipment, packaging technologies, and storage logistics that may significantly influence antimicrobial outcomes [32,41]. Greater integration between laboratory research and pilot-scale validation will therefore be important for translating experimental findings into commercially feasible food preservation strategies.

Future progress in this field will likely depend on moving beyond simplified reductionist models toward more integrative and system-oriented approaches. By incorporating matrix complexity, microbial diversity, and environmental variability, future studies may generate data that are more representative of real food systems. Improving the realism of experimental models will remain important for generating more reliable data applicable to industrial food preservation systems. Development of more realistic evaluation systems may ultimately contribute not only to improved prediction of antimicrobial efficacy but also to more effective optimization of preservation technologies suitable for industrial food applications.

## 7. Future Perspectives: From Laboratory to Industry

Despite the extensive research on the antimicrobial properties of essential oils, their large-scale application in the food industry remains relatively limited. Bridging the gap between laboratory findings and industrial implementation requires not only further scientific advances but also careful consideration of technological, regulatory, and economic factors. Although many experimental studies report promising antimicrobial outcomes, practical implementation in industrial food systems remains constrained by the complexity of real processing environments, variability in food matrices, and the need to maintain both product quality and consumer acceptance [3,25,41]. For industrial implementation, preservation strategies must remain not only microbiologically effective but also economically realistic and compatible with existing food technologies.

A major challenge concerns the scalability of strategies developed under laboratory conditions. Approaches such as encapsulation and nanoemulsion systems often demonstrate promising antimicrobial performance in experimental studies, yet their transfer to industrial production may be associated with substantial technical and economic limitations. Processing costs, formulation complexity, and compatibility with existing manufacturing technologies remain important considerations. Future work should therefore focus more strongly on developing scalable and economically feasible systems that retain the functional properties of essential oils under industrial conditions [35,36,39]. Industrial implementation additionally requires consideration of processing stability, storage compatibility, large-scale manufacturing reproducibility, and integration with existing packaging and food processing technologies [22,38,41]. Some advanced delivery systems may also involve expensive raw materials or specialized production equipment, potentially limiting their practical adoption by the food industry. For this reason, improving production efficiency and reducing formulation costs will likely become more important in future industrial development.

Regulatory requirements represent another important factor influencing industrial adoption. The approval and permitted use of essential oil compounds in foods differ considerably between regulatory agencies and geographic regions. Safety evaluation, acceptable daily intake, potential toxicity, and labeling requirements all need to be addressed before broader implementation can occur. More comprehensive toxicological evaluation and clearer regulatory frameworks will likely be necessary to support wider industrial application of essential oils in foods [39,76]. Regulatory complexity may become even more pronounced for encapsulated systems, nanoformulations, and active packaging materials containing bioactive compounds, since these technologies may require additional safety assessments regarding migration behavior, long-term exposure, and potential toxicological effects [41,76]. More harmonized international regulations could also facilitate broader commercial adoption and increase industrial confidence in essential oil-based preservation technologies.

Consumer perception is also expected to influence the future development of essential oil-based preservation systems. Although essential oils are commonly associated with natural and clean-label products, their intense aroma and flavor profiles may not be suitable for all food categories. In addition, consumer preferences can vary substantially depending on cultural background and market expectations. As a result, successful product development will require formulations that achieve antimicrobial effectiveness while maintaining acceptable sensory quality [28,39,41]. Growing consumer interest in sustainability and reduced use of synthetic additives may further support development of essential oil-based preservation systems [3,4]. Nevertheless, acceptance of such technologies will likely depend on maintaining desirable sensory quality, transparent labeling practices, and confidence in product safety and technological reliability.

From a technological standpoint, the application of essential oils should be considered alongside current trends in food innovation, including clean-label formulations and sustainable packaging materials. Multifunctional systems incorporating essential oils into biodegradable films, edible coatings, or active packaging materials may offer advantages not only in terms of microbial control but also in reducing environmental impact [21,36,41]. The integration of essential oils into multifunctional packaging systems capable of combining antimicrobial, antioxidant, and controlled-release properties may represent an important future direction for food preservation technologies [32,34,67,68,69,70]. In addition, biodegradable and bio-based packaging materials incorporating natural antimicrobial compounds may contribute to reducing environmental burdens associated with conventional synthetic packaging systems.

Emerging technologies such as nanotechnology, smart packaging systems, and AI-assisted formulation approaches are creating new opportunities for optimizing essential oil applications in foods [37,38,75]. Predictive modeling, machine learning, and data-driven optimization tools are increasingly used to identify more effective combinations of essential oils, delivery systems, packaging materials, and storage conditions suitable for specific food products and industrial applications.

Closer collaboration between academic researchers and the food industry will also be necessary for accelerating practical implementation. Interdisciplinary approaches involving food scientists, microbiologists, chemists, and engineers may contribute to the development of more effective and commercially feasible preservation systems. In particular, pilot-scale validation and industrial-scale studies remain necessary to confirm whether results obtained under laboratory conditions can be reproduced in real manufacturing environments [35,36,41]. Closer interaction between academia, industry, and regulatory agencies may additionally support the development of standardized evaluation methods, harmonized safety assessments, and more efficient translation of experimental findings into commercial food technologies. Expanding pilot-scale and industrial validation studies will therefore be essential for reducing the gap between laboratory research and practical implementation.

The future use of essential oils in the food industry will likely depend on balancing antimicrobial efficacy with technological feasibility, regulatory compliance, economic considerations, and consumer expectations. Addressing these interconnected challenges will be important for expanding the practical application of essential oils as natural food preservatives. Successful industrial application of essential oils will require multidisciplinary approaches capable of addressing microbiological safety, physicochemical stability, sensory quality, sustainability, and commercial feasibility simultaneously.

## 8. Conclusions

Essential oils have attracted considerable attention as natural antimicrobial agents with potential applications in food preservation. Although strong antimicrobial activity is frequently reported under in vitro conditions, translating these findings into real food systems remains a substantial challenge. The complexity of food matrices, together with physicochemical instability, sensory limitations, and microbial adaptation, can markedly reduce the antimicrobial performance of essential oils under practical conditions. For this reason, antimicrobial efficacy should be interpreted within the broader context of food composition, environmental conditions, microbial ecology, and technological feasibility rather than relying solely on simplified laboratory observations.

This review demonstrates that the discrepancy between in vitro activity and efficacy in food systems is largely associated with the limitations of simplified experimental models. The proposed “Five-Barrier Framework for Essential Oil Efficacy in Food Systems” provides a structured approach for understanding the interacting factors that influence antimicrobial activity in complex food environments. By integrating factors such as partitioning behavior, matrix interactions, physicochemical instability, sensory constraints, and microbial adaptation, the framework offers a more realistic perspective for evaluating essential oil performance in practical food systems.

Current evidence suggests that improving the practical applicability of essential oils will require more realistic and system-oriented research strategies. Approaches such as encapsulation technologies, synergistic combinations, and active packaging systems may help overcome several of the barriers limiting antimicrobial efficacy. In parallel, the use of more representative evaluation models is necessary to obtain data that are more relevant to real food applications. Advanced delivery systems, controlled-release technologies, and multifunctional packaging materials could further improve antimicrobial stability, reduce sensory limitations, and increase compatibility with industrial food preservation practices.

Further progress in this field will depend on balancing antimicrobial effectiveness with technological feasibility, regulatory requirements, economic considerations, and consumer acceptance. Closer integration between laboratory research and industrial application will therefore be essential for supporting broader use of essential oils as natural food preservatives. Future research should also place greater emphasis on pilot-scale validation, industrial feasibility studies, regulatory harmonization, and standardized evaluation methods capable of improving reproducibility and practical relevance of antimicrobial assessments.

Despite existing limitations, essential oils remain promising candidates for development of alternative preservation strategies. Future advances will depend not only on continued technological innovation but also on improved understanding of essential oil behavior in complex food systems. Practical implementation of essential oils in food preservation will require multidisciplinary approaches integrating microbiology, food science, chemistry, packaging technology, sensory analysis, and industrial processing considerations. Such multidisciplinary approaches could help develop preservation systems that are safer, more sustainable, and better accepted by consumers.

## Figures and Tables

**Table 1:** Summary of key barriers affecting the antimicrobial efficacy of essential oils in food systems.

Barrier	Mechanism	Impact on EO Efficacy	Example Food System
Partitioning	Distribution of essential oils into the lipid phase	Reduced availability in the aqueous phase where microorganisms are present	High-fat foods
Binding interactions	Interactions with proteins and carbohydrates	Reduced bioavailability and antimicrobial activity	Dairy systems
Instability	Oxidation, evaporation, and thermal degradation	Loss of active volatile compounds during processing and storage	Thermally processed foods
Sensory limitations	Strong aroma and flavor profiles	Restriction of applicable concentrations due to consumer acceptability	Dairy and seafood products
Microbial adaptation	Biofilm formation and stress-response mechanisms	Increased microbial tolerance and resistance	Mixed microbial cultures

EO, essential oil.

**Table 2:** Practical consequences of the major barriers affecting essential oil efficacy in food systems.

Barrier	Consequence in Food Systems	Practical Example
Food matrix interactions	Reduced availability of active compounds due to interactions with lipids, proteins, and carbohydrates	Meat and dairy products
Physicochemical instability	Loss of active constituents during processing and storage	Thermally processed foods
Sensory constraints	Limited usable concentration because of changes in flavor and aroma	Dairy and seafood products
Microbial behavior	Increased microbial tolerance and reduced susceptibility	Food-processing environments

**Table 3:** Representative studies evaluating the discrepancy between in vitro antimicrobial activity and efficacy of essential oils in meat and meat products.

Essential Oil	Target Microorganism	In Vitro Activity	Meat Matrix	EO Concentration in Food	Effect In Food System	Main Limitation	Ref.
Oregano	*Listeria monocytogenes*	MIC = 0.18 µL/mL	Minced pork meat	0.18–0.36 µL/g	0.25–0.51 log_10_ CFU/g ↓ (4 d, 4 °C)	Matrix effects	[19]
Thyme	*Listeria monocytogenes*	MIC = 0.36 µL/mL	Minced pork meat	0.36–0.72 µL/g	0.22–0.29 log_10_ CFU/g ↓ (4 d, 4 °C)	Matrix effects	[19]
Thyme	*Yersinia enterocolitica*	MIC = 0.23 µL/mL	Minced pork meat	0.23–0.46 µL/g	0.38–0.64 log_10_ CFU/g ↓	Matrix effects	[47]
Oregano	*Yersinia enterocolitica*	MIC = 0.09–0.18 µL/mL	Minced pork meat	0.09–0.18 µL/g	No significant reduction	Matrix protection	[47]
Cinnamon	*Staphylococcus aureus*	Strong in vitro activity	Minced beef	1.5%	5.56 log_10_ CFU/g ↓ (3 h)	High concentration required	[48]
Cinnamon	*Escherichia coli*	Strong in vitro activity	Minced beef	1.5%	3.58 log_10_ CFU/g ↓ (3 h)	Reduced food-system efficacy	[48]
Clove	*Staphylococcus aureus*	Strong in vitro activity	Minced beef	1.5%	4.01 log_10_ CFU/g ↓ (3 h)	Sensory constraints	[48]
Rosemary	*Staphylococcus aureus*	Moderate in vitro activity	Minced beef	1.5%	2.84 log_10_ CFU/g ↓ (3 h)	Lower efficacy in meat matrix	[48]
*Origanum floribundum* EO	*Salmonella Typhimurium*	MIC determined	Minced meat	5×–20× MIC	Significant reduction only at multiple MIC levels	Elevated concentrations required	[49]
*Thymus pallescens* EO	*Salmonella Typhimurium*	MIC determined	Minced meat	5×–20× MIC	Improved microbial control under refrigeration	Above-MICs required	[49]

**Abbreviations:** EO, essential oil; MIC, minimum inhibitory concentration; CFU, colony-forming units; ↓, reduction.

**Table 4:** Comparative antimicrobial efficacy of essential oils in dairy products.

Essential Oil	Target Microorganism(s)	Dairy Matrix	EO Concentration in Food System	Effect in Food System	Main Limitation	Ref.
OEO	Total spoilage microbiota	Pasteurized milk	2.0 mg/L	TPC below regulatory limit throughout 8 d storage	Matrix interactions	[44]
OEO	*E. coli* O157:H7	Feta cheese	0.5% in sodium alginate film	No significant reduction	Limited diffusion	[43]
OEO	*L. monocytogenes*	Feta cheese	0.5% in sodium alginate film	Survival reduced: 28 → 17 d (4 °C); 28 → 10 d (12 °C)	Storage-dependent efficacy	[43]
OEO	Spoilage microbiota	Feta cheese	0.5% in sodium alginate film	Shelf life +4 d (4 °C); +7 d (12 °C)	Moderate impact on spoilage microbiota	[43]
*T. spicata* EO	*E. coli*, *S. aureus*, *B. subtilis*, *P. aeruginosa*	Curd cheese	1–5% nanoemulsion	Strong in vitro activity (29.8–34.0 mm); no measurable antibacterial effect in cheese; spoilage delayed	Stability ≠ efficacy	[50]
REO	*L. monocytogenes*, *E. coli*, *S. aureus*, *P. aeruginosa*	White soft cheese	0.6–1.0% in WPC coating	Total bacterial counts: 6.35 → 1.50 log CFU/g (28 d)	Coating-dependent delivery	[51]
REO	Coliform bacteria	White soft cheese	1.0% in WPC coating	Counts: 6.33 → 1.53 log CFU/g (28 d)	Coating-dependent delivery	[51]
REO	Yeasts and molds	White soft cheese	1.0% in WPC coating	Counts: 6.68 → 1.70 log CFU/g (28 d)	Coating-dependent delivery	[51]

**Abbreviations:** OEO, oregano essential oil; REO, rosemary essential oil; TPC, total plate count; WPC, whey protein concentrate.

**Table 6:** Strategies to enhance the efficacy of essential oils in food applications.

Strategy	Principle	Advantages	Limitations
Encapsulation	Protection of essential oil compounds within carrier systems	Improved physicochemical stability and controlled release	Increased production cost
Nanoemulsions	Enhancement of dispersion and solubility in food matrices	Increased bioavailability and antimicrobial efficacy	Formulation complexity and stability challenges
Combination approaches	Synergistic interactions with other preservation methods or antimicrobials	Reduced required dose and enhanced antimicrobial activity	Potential interaction effects and formulation variability
Active packaging	Controlled release of essential oils from packaging materials	Extended shelf life and prolonged antimicrobial protection	Increased packaging and production costs

## Data Availability

No new data were created or analyzed in this study.

## Abbreviations

The following abbreviations are used in this manuscript:

EO	Essential oil
EOs	Essential oils
MIC	Minimum inhibitory concentration
MBC	Minimum bactericidal concentration
CFU	Colony-forming units
OEO	Oregano essential oil
REO	Rosemary essential oil
TPC	Total plate count
WPC	Whey protein concentrate
TVB-N	Total volatile basic nitrogen
TBA	Thiobarbituric acid reactive substances
EGCG	Epigallocatechin gallate
WSEO	Wampee seed essential oil
NE	Nanoemulsion
NLC	Nanostructured lipid carriers
